# Multimorbidity Patterns in the Elderly: A New Approach of Disease Clustering Identifies Complex Interrelations between Chronic Conditions

**DOI:** 10.1371/journal.pone.0015941

**Published:** 2010-12-29

**Authors:** Ingmar Schäfer, Eike-Christin von Leitner, Gerhard Schön, Daniela Koller, Heike Hansen, Tina Kolonko, Hanna Kaduszkiewicz, Karl Wegscheider, Gerd Glaeske, Hendrik van den Bussche

**Affiliations:** 1 Department of Primary Medical Care, University Medical Center Hamburg-Eppendorf, Hamburg, Germany; 2 Department of Medical Biometry and Epidemiology, University Medical Center Hamburg-Eppendorf, Hamburg, Germany; 3 Division of Health Economics, Health Policy and Health Services Research, Centre for Social Policy Research, University of Bremen, Bremen, Germany; Yale University School of Medicine, United States of America

## Abstract

**Objective:**

Multimorbidity is a common problem in the elderly that is significantly associated with higher mortality, increased disability and functional decline. Information about interactions of chronic diseases can help to facilitate diagnosis, amend prevention and enhance the patients' quality of life. The aim of this study was to increase the knowledge of specific processes of multimorbidity in an unselected elderly population by identifying patterns of statistically significantly associated comorbidity.

**Methods:**

Multimorbidity patterns were identified by exploratory tetrachoric factor analysis based on claims data of 63,104 males and 86,176 females in the age group 65+. Analyses were based on 46 diagnosis groups incorporating all ICD-10 diagnoses of chronic diseases with a prevalence ≥ 1%. Both genders were analyzed separately. Persons were assigned to multimorbidity patterns if they had at least three diagnosis groups with a factor loading of 0.25 on the corresponding pattern.

**Results:**

Three multimorbidity patterns were found: 1) cardiovascular/metabolic disorders [prevalence female: 30%; male: 39%], 2) anxiety/depression/somatoform disorders and pain [34%; 22%], and 3) neuropsychiatric disorders [6%; 0.8%]. The sampling adequacy was meritorious (Kaiser-Meyer-Olkin measure: 0.85 and 0.84, respectively) and the factors explained a large part of the variance (cumulative percent: 78% and 75%, respectively). The patterns were largely age-dependent and overlapped in a sizeable part of the population. Altogether 50% of female and 48% of male persons were assigned to at least one of the three multimorbidity patterns.

**Conclusion:**

This study shows that statistically significant co-occurrence of chronic diseases can be subsumed in three prevalent multimorbidity patterns if accounting for the fact that different multimorbidity patterns share some diagnosis groups, influence each other and overlap in a large part of the population. In recognizing the full complexity of multimorbidity we might improve our ability to predict needs and achieve possible benefits for elderly patients who suffer from multimorbidity.

## Introduction

Multimorbidity is a common problem in the elderly and its occurrence rises with age. Medicare data suggest that 32% of the population in the age group 65–69 suffer from three or more chronic conditions. In the age group 80–84 the prevalence of multimorbidity increased to 52% [1]. 76% of the patients in general practice have three or more chronic conditions [2]. Multimorbidity is significantly associated with higher mortality, increased disability, a decline of functional status and a lower quality of life. It also leads to a greater extent of health care utilization (costs, length of hospital stay, and number of physician visits) [3]. The general practitioner (GP) has little help in adjusting care for multiple chronic conditions, because clinical practice guidelines are mostly focused on one disease only. Adhering to current clinical practice guidelines in the treatment of multimorbidity may therefore even have adverse effects [4]. Information about the specific elements and processes in multimorbidity, the interactions and possible synergies of the diseases is urgently needed in order to facilitate diagnosis, amend prevention, lower costs in health care systems and increase the patients' quality of life.

According to Schellevis, multimorbidity may be classified by the relationship between the different diseases. Concurrent comorbidity defines the random coexistence of diseases. Cluster comorbidity indicates statistically significant associations between diseases without a causal explanation. Causal comorbidity describes disease clustering with a pathophysiological relation between the different diseases (e.g. shared risk factors). And finally, complicating comorbidity illustrates the case when one disease is caused by another disease and cannot be explained without its precursor [5].

If we try to understand the distribution of diseases in multimorbidity we face a labyrinth full of possibilities. All diseases are more or less statistically associated with each other. So if we start at any point of this labyrinth and we have no guide to find our way, we might easily get lost. For that reason, it seams important to discover the underlying structure in the distribution of disease combinations, i.e. which pathways may lead through the labyrinth of multimorbidity.

The aim of this paper is therefore to separate concurrent (random) comorbidity from significantly associated comorbidity by identifying multimorbidity patterns in the distribution of chronic diseases and to analyze age and gender-specific differences in the patterns.

## Methods

The analyses are based on ambulatory data of the Gmünder ErsatzKasse, a German statutory health insurance company with 1.7 million insurants (in 2008), which corresponds to 2.4% of the statutory insured population [6]. The Gmünder ErsatzKasse has a greater proportion of male insurants in the elderly than in the general population of Germany therefore data analyses have to be adjusted for gender. The dataset contains pseudonymous data from every insured member of this company.

The sample for our analyses consists of all persons aged 65 years and older who were permanently insured during the year 2006. For the distribution of age, gender and the number of diagnosis groups in the sample cf. table 1. The analysis of morbidity was based on a list of 46 defined diagnosis groups of chronic diseases (see below) based on ICD-10 (International Statistical Classification of Diseases and Related Health Problems, 10th Revision) codes. The diagnoses were only counted if they were coded in at least three out of four quarters (three month periods) within the year 2006. This criterion was chosen in order to increase the validity of the diagnoses based on claims data by avoiding transitory or even accidental diagnoses. For prevalence, gender-specific rank order and ICD-10 codes of the diagnosis groups cf. table 2.

**Table 1:** Sample size, age and number of diagnosis groups by gender.

	Female	Male
**n (% of total population)**	63 104 (42.3)	86 176 (57.7)
**Mean age (sd)**	72.6 (6.6)	71.4 (5.7)
**Mean number of diagnosis groups (sd)**	4.4 (3.3)	4.0 (3.3)

**Table 2:** Prevalence of diagnosis groups (in %) and rank order for female and male.

	Diagnosis group [ICD-10 codes]	Female (rank)	Male (rank)
1	Hypertension [I10-15]	54.8 (1)	51.2 (1)
2	Lipid metabolism disorders [E78]	34.6 (2)	32.7 (2)
3	Chronic low back pain [M40-45, M47, M48.0-.2, M48.5-.9, M50-54]	34.6 (3)	28.2 (3)
4	Diabetes mellitus [E10-14]	18.3 (6)	21.7 (4)
5	Joint arthrosis [M15-19]	24.4 (4)	16.6 (7)
6	Chronic ischemic heart diseases [I20-21, I25]	13.0 (11)	21.5 (5)
7	Thyroid dysfunction [E01-05, E06.1-.3, E06.5, E06.9, E07]	23.1 (5)	9.3 (14)
8	Severe vision reduction [H17-18, H25-28, H31, H33, H34.1-.2, H34.8-.9, H35-36, H40, H43, H47, H54]	16.5 (7)	13.9 (10)
9	Cancers [C00-26, C30-41, C43-58, C60-97, D00-09, D37-48]	11.3 (13)	15.0 (9)
10	Cardiac arrhythmias [I44-45, I46.0, I46.9, I47-48, I49.1-.9]	11.2 (14)	13.4 (11)
11	Purine/pyrimidine metabolism disorders/Gout [E79, M10]	7.9 (17)	15.6 (8)
12	Lower limb varicosis [I83, I87.2]	16.2 (8)	7.6 (16)
13	Prostatic hyperplasia [N40]		19.3 (6)
14	Asthma/Chronic obstructive pulmonary disease [J40-45, J47]	10.3 (15)	11.3 (12)
15	Atherosclerosis/Peripheral arterial occlusive disease [I65-66, I67.2, I70, I73.9]	7.3 (19)	10.7 (13)
16	Depression [F32-33]	13.0 (10)	5.2 (23)
17	Obesity [E66]	9.4 (16)	7.6 (17)
18	Liver diseases [K70, K71.3-.5, K71.7, K72.1, K72.7, K72.9, K73-74, K76]	7.0 (21)	9.0 (15)
19	Osteoporosis [M80-82]	14.4 (9)	2.4 (35)
20	Chronic gastritis/Gastroesophageal reflux disease [K21, K25.4-.9, K26.4-.9, K27.4-.9, K28.4-.9, K29.2-.9]	7.8 (18)	7.2 (18)
21	Cerebral ischemia/Chronic stroke [G45, I60-64, I69]	5.3 (27)	6.9 (19)
22	Cardiac insufficiency [I50]	7.2 (20)	5.3 (22)
23	Neuropathies [G50-64]	5.7 (24)	5.4 (20)
24	Noninflammatory gynecological problems [N81, N84-90, N93, N95]	12.6 (12)	
25	Chronic cholecystitis/Gallstones [K80, K81.1]	6.6 (22)	4.0 (28)
26	Allergies [H01.1, J30, K52.2, K90.0, L23, L27.2, L56.4, T78.1, T78.4, T88.7]	6.1 (23)	4.2 (25)
27	Insomnia [F51, G47]	5.4 (26)	3.8 (29)
28	Renal insufficiency [N18-N19]	2.9 (36)	5.3 (21)
29	Intestinal diverticulosis [K57]	4.5 (29)	4.0 (26)
30	Hemorrhoids [I84]	3.7 (34)	4.5 (24)
31	Somatoform disorders [F45]	5.5 (25)	2.9 (32)
32	Cardiac valve disorders [I34-I37]	3.7 (33)	4.0 (27)
33	Urinary incontinence [N39.3-.4, R32]	5.1 (28)	2.3 (37)
34	Severe hearing loss [H90, H91.0-.1, H91.3, H91.8-.9]	2.8 (37)	3.5 (30)
35	Dementias [F00-03, F05.1, G30-31, R54]	4.0 (30)	2.6 (34)
36	Dizziness [H81-82, R42]	3.8 (32)	2.2 (38)
37	Rheumatoid arthritis/Chronic polyarthritis [M05-06, M79.0]	3.9 (31)	1.7 (40)
38	Urinary tract calculi [N20]	1.4 (42)	3.2 (31)
39	Anemias [D50-53, D55-58, D59.0-.2, D59.4-.9, D60.0, D60.8-.9, D61, D63-64]	2.5 (39)	2.4 (36)
40	Migraine/Chronic headache [G43-44]	3.6 (35)	1.2 (43)
41	Psoriasis [L40]	1.6 (40)	2.1 (39)
42	Anxiety [F40-41]	2.6 (38)	1.1 (44)
43	Sexual dysfunction [F52, N48.4]		2.9 (33)
44	Parkinson's disease [G20-22]	1.2 (43)	1.4 (42)
45	Tobacco abuse [F17]	0.8 (44)	1.6 (41)
46	Hypotension [I95]	1.6 (41)	0.9 (45)

The methods for compiling the list of 46 diagnosis groups have been described elsewhere in detail [7]. In short, we used the most frequent conditions in GP surgeries as mentioned in a panel survey of the Central Research Institute of Statutory Ambulatory Health Care in Germany (“ADT-Panel”) [8]. Chronicity of diagnoses was assessed using the scientific expert report for the formation of a morbidity orientated risk adjustment scheme in the German Statutory Health Insurance [9]. In order to capture a comprehensive picture of the disease patterns in individual patients we amended this list for all chronic conditions with a prevalence ≥1% in the age group ≥65 years in the data set of the Gmünder ErsatzKasse in 2006. ICD-10 codes were grouped together if diseases and syndromes had a close pathophysiological similarity and if ICD codes of related disorders were used ambiguously by coding physicians in clinical reality, respectively.

The research expressed in our article was conducted according to the principles expressed in the Declaration of Helsinki. We did not have to obtain informed consent, because our research was based on insurance claims data and the data set was analyzed anonymously (as regulated by German law in §75 SGB X). The study was approved by the Ethics Committee of the Medical Association of Hamburg including the waiver of consent (approval no. PV3057).

### Statistical Analyses

Correlations between diagnosis groups were analyzed by exploratory factor analysis. We chose the principal factor method, because we presumed that the factors would not explain the whole variance of the analyzed diagnosis groups. We used a tetrachoric correlation matrix for factor analysis, which is supposed to be an appropriate method for dichotomous data [10]. In doing so, we made the assumption that the dichotomous diagnoses reflect an underlying continuous latent trait. In other words: we presumed that the chronic diseases included in our analysis have a progressive course (i.e. cumulation of risk factors before onset and/or progression after onset) and they get diagnosed if in this course a certain threshold is reached. The factors that result from our analysis can be interpreted as multimorbidity patterns (i.e. clusters of diagnosis groups frequently associated with each other) and each factor loading represents the association of the specific diagnosis group with a pattern. As a measure of model fit we also reported cumulative percent which describes the proportion of variance of the diagnosis data that can be explained be the patterns. The sampling adequacy for performing a factor analysis was confirmed by assessing the Kaiser-Meyer-Olkin measure for data of both genders.

Diagnosis groups of male and female patients were analyzed separately. Gender-specific diagnoses for female patients were excluded in analyses of male patients and vice versa (cf. table 2). Factors were regarded as substantial if they had a minimum eigenvalue of 1.0. A variable was defined to be associated with a factor if it had a factor loading of 0.25 or more. We defined a relatively permissive threshold for factor loadings because we expected a large amount of random associations (i.e. concurrent comorbidity) between the diagnosis groups. Factors were allowed to be associated with each other, i.e. we assumed that being in one multimorbidity pattern may influence the risk of being in another pattern as well. For this reason we used an oblique (oblimin) rotation of factor loading matrices.

For the calculation of prevalences of multimorbidity patterns we assigned individual patients to a pattern if they had diagnoses in at least three groups with a factor loading of 0.25 on the corresponding pattern. For analyses of pattern prevalences negative factor loadings were considered non-instructive and ignored. Prevalence figures were created using calculated values and a smoothing plot using lowess prediction with a smoother span of 0.5. Because of the low number of cases in the age groups ≥90, age-specific prevalence rates for these age groups were considered as non-informative and excluded from these figures.

Data preparation was done with SAS (Version 9.2). Statistical analyses were made with Stata/MP (version 11.0) and figures were created using R (version 2.12.0).

## Results

### Female Patients

For female patients we assessed a Kaiser-Meyer-Olkin measure of 0.84 which corresponds to a meritorious sampling adequacy of the data. Factor analysis of diagnosis groups of female patients resulted in the emergence of three factors (cf. table 3) with a cumulative percent of 78.0. The first factor can be interpreted as multimorbidity pattern of cardiovascular and metabolic disorders [eigenvalue 6.34]. The second factor was named anxiety, depression, somatoform disorders (ADS) and pain [2.37]. As a third pattern we identified neuropsychiatric disorders [1.71].

**Table 3:** Loadings of factors with eigenvalue ≥1 in diagnosis groups of female patients.

	CMD	ADS/P	NPS
**Eigenvalue**	**6.34**	**2.37**	**1.71**
**Cumulative percent**	**47.5**	**65.2**	**78.0**
- Hypertension	.71		
- Lipid metabolism disorders	.45		
- Chronic low back pain		.61	
- Diabetes mellitus	.55		
- Joint arthrosis		.35	
- Chronic ischemic heart diseases	.38		.31
- Thyroid dysfunction		.27	
- Cardiac arrhythmias	.28		
- Purine/pyrimidine metabolism disorders/Gout	.60		
- Lower limb varicosis		.31	
- Asthma/Chronic obstructive pulmonary disease		.27	
- Atherosclerosis/Peripheral arterial occlusive disease	.35		.25
- Depression		.44	.36
- Obesity	.52		
- Liver diseases	.39		
- Osteoporosis		.37	
- Chronic gastritis/Gastroesophageal reflux disease		.36	
- Cerebral ischemia/Chronic stroke			.43
- Cardiac insufficiency	.26		.51
- Noninflammatory gynecological problems		.52	
- Chronic cholecystitis/Gallstones	.29		
-Allergies		.38	
- Insomnia		.31	
- Renal insufficiency	.47		.33
- Intestinal diverticulosis		.32	
- Hemorrhoids		.45	
- Somatoform disorders		.51	
- Cardiac valve disorders	.31		
- Urinary incontinence			.49
- Dementias			.78
- Dizziness		.30	.30
- Rheumatoid arthritis/Chronic Polyarthritis		.29	
- Urinary tract calculi	.28		
- Anemias			.29
- Migraine/Chronic headache		.53	
- Anxiety		.39	
- Parkinson's disease			.56
- Hypotension	−.38	.58	

**Factor loadings <.25 have been omitted. CMD: cardiovascular and metabolic disorders; ADS/P: anxiety, depression, somatoform disorders and pain; NPS: neuropsychiatric disorders.**

**Figure 1:**
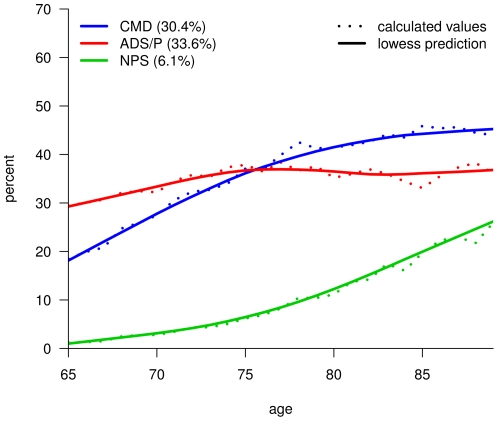
Prevalence by age for multimorbidity patterns of female patients. CMD: cardiovascular and metabolic disorders; ADS/P: anxiety, depression, somatoform disorders and pain; NPS: neuropsychiatric disorders.

The age dependency of these multimorbidity patterns is shown in figure 1. Prevalence rates of the cardiovascular/metabolic (30.4% total prevalence; 18.5% in 65 years old patients to 44.0% in 89 years old patients) and neuropsychiatric patterns (6.1%; 1.3% to 25.9%) significantly increase with age while the ADS and pain pattern increases only slightly (33.6%; 29.5% to 36.1%).

**Figure 2:**
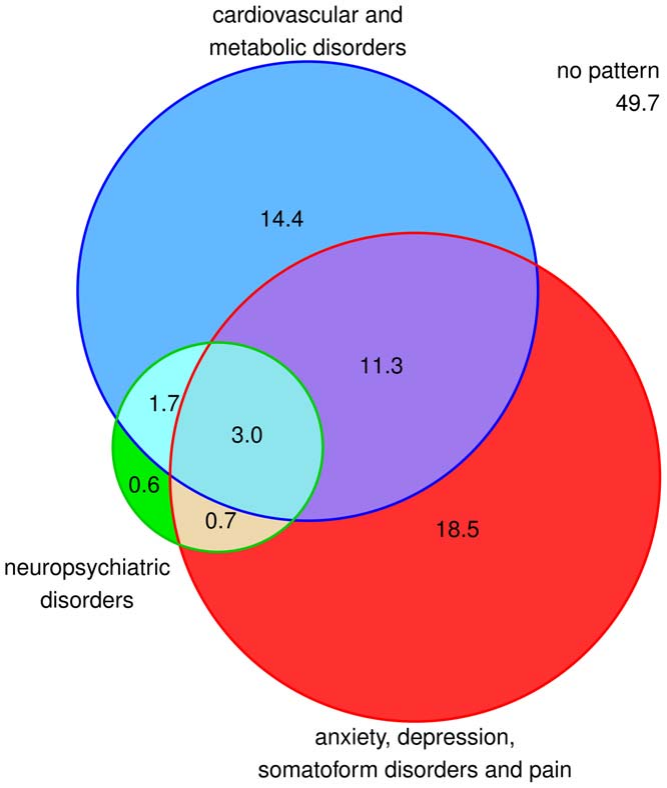
Overlapping of multimorbidity patterns (in %) related to the total female population.

In total 50.3% of the female patients in our sample can be attributed to one or more multimorbidity patterns. The overlapping of patterns related to the total female population is shown in figure 2. 14.4% of all female patients are exclusively attributed to cardiovascular/metabolic disorders and no other pattern, 18.5% exclusively to ADS and pain and 0.6% exclusively to neuropsychiatric disorders. 11.3% of the female patients overlap between cardiovascular/metabolic disorders and ADS and pain, 1.7% between cardiovascular/metabolic and neuropsychiatric disorders and 0.7% between ADS and pain and neuropsychiatric disorders. An overlapping between all three patterns can be found in 3.0% of the female patients.

**Figure 3:**
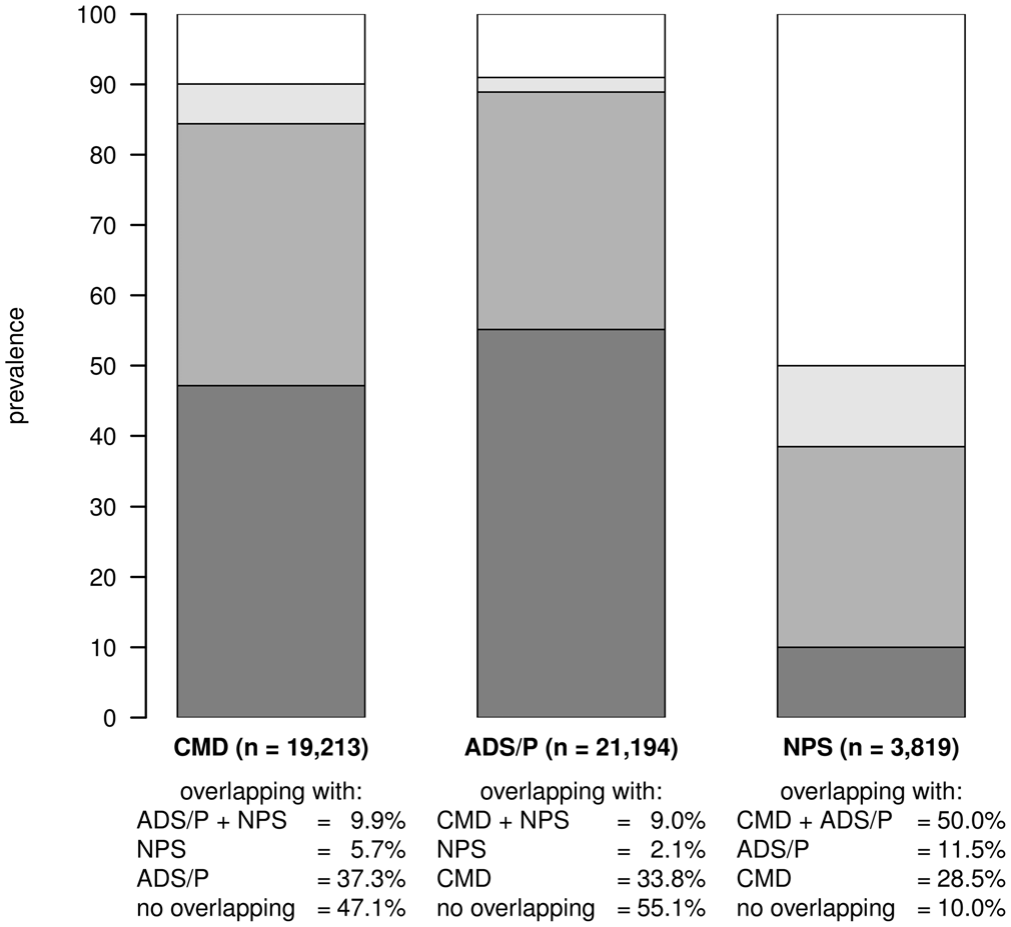
Proportional overlapping of each pattern related to the other patterns for female patients. CMD: cardiovascular and metabolic disorders; ADS/P: anxiety, depression, somatoform disorders and pain; NPS: neuropsychiatric disorders.

For female patients the proportional overlapping of each pattern related to the other patterns is shown in figure 3. 47.1% and 55.1% of all female patients with ADS and pain and cardiovascular/metabolic disorders, respectively, are only attributed to this and no other pattern, while another 37.3% and 33.8%, respectively, are assigned to both patterns simultaneously. Only 10.0% of all female patients with neuropsychiatric disorders refer to no other pattern, while 28.5% overlap with the cardiovascular/metabolic disorders and 11.5% with ADS and pain, respectively. 50.0% of neuropsychiatric patients are also assigned to cardiovascular/metabolic disorders and ADS and pain at the same time.

### Male patients

For male patients we assessed a Kaiser-Meyer-Olkin measure of 0.85, which also corresponds to a meritorious sampling adequacy. Factor analysis of diagnosis groups of male patients resulted in the emergence of three factors (cf. table 4) with a total of 75.3 cumulative percent. The first factor can be interpreted as multimorbidity pattern of ADS and pain [7.14]. As a second pattern we identified cardiovascular and metabolic disorders [1.96]. The third factor can be named as neuropsychiatric disorders [1.75].

**Table 4:** Loadings of factors with eigenvalue ≥1 in diagnosis groups of male patients.

	ADS/P	CMD	NPS
**Eigenvalue**	**7.14**	**1.96**	**1.75**
**Cumulative percent**	**49.5**	**63.1**	**75.3**
Hypertension		.70	
Lipid metabolism disorders		.49	
Chronic low back pain	.66		
Diabetes mellitus		.59	
Joint arthrosis	.44		
Chronic ischemic heart diseases		.53	
Thyroid dysfunction	.27		
Cardiac arrhythmias		.37	
Purine/pyrimidine metabolism disorders/Gout		.50	
Lower limb varicosis	.33		
Prostatic hyperplasia	.43		
Asthma/Chronic obstructive pulmonary disease	.27		
Atherosclerosis/Peripheral arterial occlusive disease		.50	
Depression	.47		.33
Obesity		.47	
Liver diseases		.40	
Osteoporosis	.35		
Chronic gastritis/Gastroesophageal reflux disease	.40		
Cerebral ischemia/Chronic stroke		.30	.38
Cardiac insufficiency		.46	.31
Neuropathies		.27	
Chronic cholecystitis/Gallstones		.25	
Allergies	.41		
Insomnia	.37		
Renal insufficiency		.55	
Intestinal diverticulosis	.40		
Hemorrhoids	.51		
Somatoform disorders	.58		
Cardiac valve disorders		.37	
Urinary incontinence			.55
Severe hearing loss	.27		
Dementias			.70
Dizziness	.37		
Urinary tract calculi	.30		
Anemias		.30	
Migraine/Chronic headache	.49		
Anxiety	.47		
Sexual dysfunction	.35		
Parkinson's disease			.60
Tobacco abuse		.32	
Hypotension	.54	−.27	

**Factor loadings <.25 have been omitted. CMD: cardiovascular and metabolic disorders; ADS/P: anxiety, depression, somatoform disorders and pain; NPS: neuropsychiatric disorders.**

**Figure 4:**
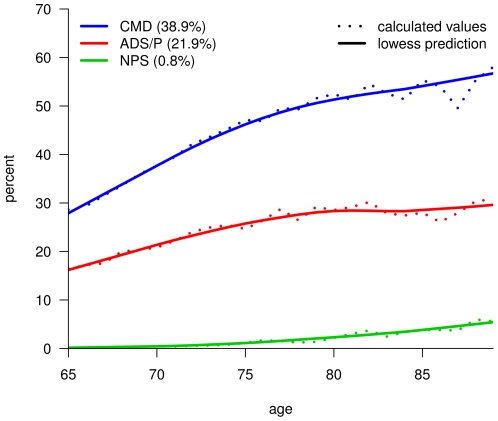
Prevalence by age for multimorbidity patterns of male patients. CMD: cardiovascular and metabolic disorders; ADS/P: anxiety, depression, somatoform disorders and pain; NPS: neuropsychiatric disorders.

The age dependency of these multimorbidity patterns is shown in figure 4. The prevalence rates of all three patterns significantly increase with age, i.e. the cardiovascular/metabolic pattern [total prevalence: 38.9%] from 28.6% in 65 years old patients to 58.0% in 89 years old patients, the ADS and pain pattern [21.9%] from 16.5% to 30.8% and the neuropsychiatric pattern [0.8%] from 0.1% to 5.6%.

**Figure 5:**
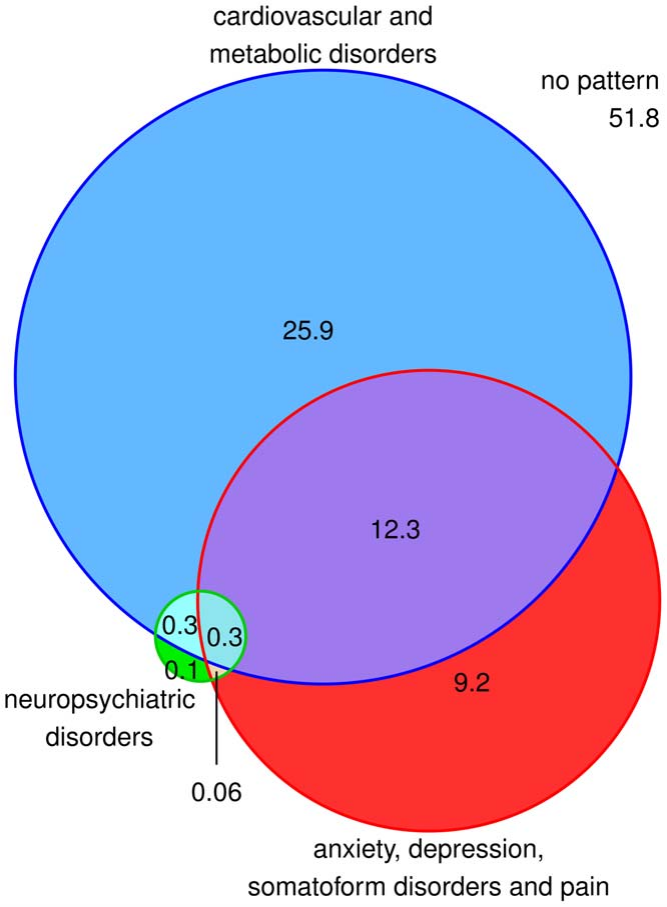
Overlapping of multimorbidity patterns (in %) related to the total male population.

In total 48.2% of male patients are at least assigned to one multimorbidity pattern. The overlapping of patterns related to the total male population is shown in figure 5. 25.9% of all male patients are exclusively attributed to cardiovascular/metabolic disorders, 9.2% exclusively to ADS and pain and 0.1% exclusively to NPS. 12.3% of the male patients overlap between cardiovascular/metabolic disorders and ADS and pain, 0.3% between cardiovascular/metabolic and neuropsychiatric disorders and 0.06% between ADS and pain and neuropsychiatric disorders. An overlapping between all three patterns can be found in 0.3% of the male patients.

**Figure 6:**
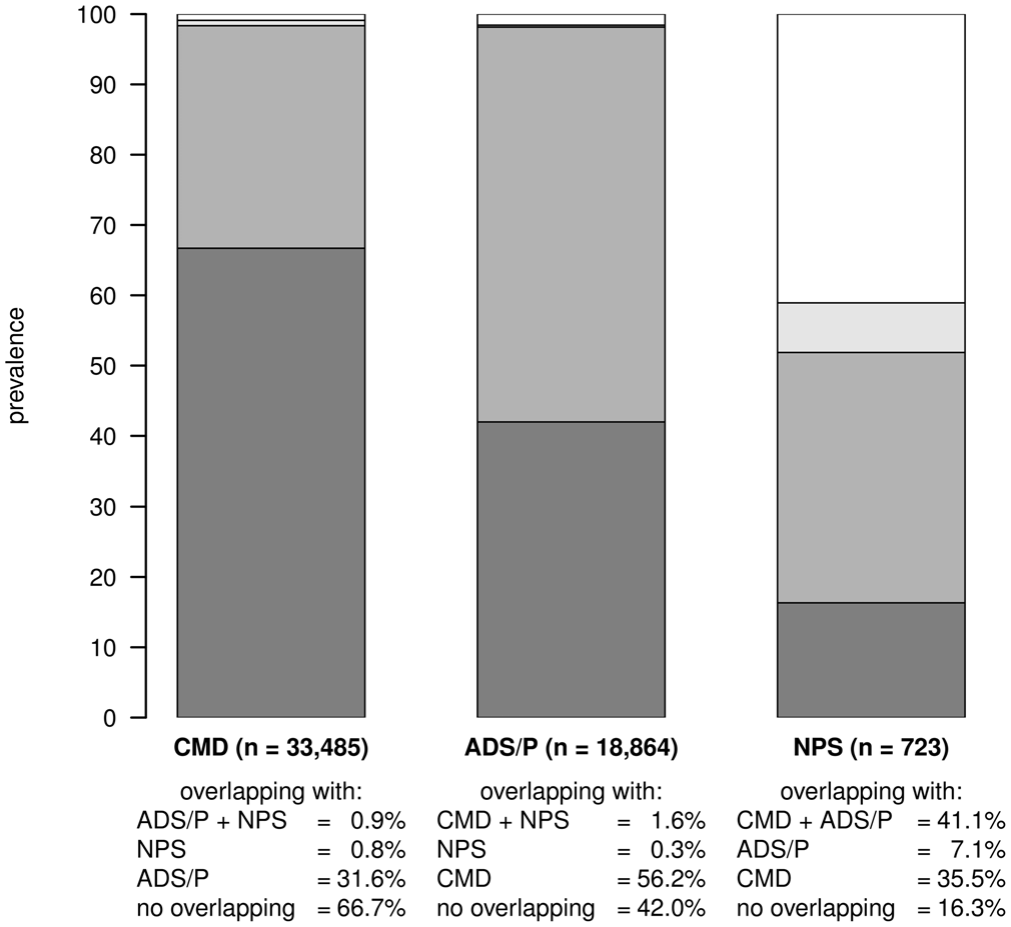
Proportional overlapping of each pattern related to the other patterns for male patients. CMD: cardiovascular and metabolic disorders; ADS/P: anxiety, depression, somatoform disorders and pain; NPS: neuropsychiatric disorders.

For male patients the proportional overlapping of each pattern related to the other patterns is shown in figure 6. Similar to the pattern structure of female patients, a large part of patients with cardiovascular/metabolic disorders (66.7%) and ADS and pain (42.0%) are only attributed to this and no other pattern while 31.6% and 56.2%, respectively, simultaneously refer to both patterns. 16.3% of male patients with neuropsychiatric disorders are assigned to this and no other pattern, while 7.1% are also attributed to ADS and pain, 35.5% to neuropsychiatric and cardiovascular/metabolic disorders and 41.1% refer at the same time to ADS and pain, neuropsychiatric as well as cardiovascular/metabolic disorders.

There are considerable differences between the male and female sample regarding the composition of the multimorbidity patterns. The biggest gender differences are in the neuropsychiatric pattern, which includes chronic ischemic heart diseases, atherosclerosis/peripheral arterial occlusive disease, dizziness, renal insufficiency and anemia only in the female population. The cardiovascular/metabolic pattern includes urinary tract calculi only for female patients while in this pattern chronic stroke, neuropathies, anemia and tobacco abuse are only present for male patients. Finally, the ADS and pain pattern includes noninflammatory gynecological problems and rheumatoid arthritis only for female as well as prostatic hyperplasia, severe hearing loss, urinary tract calculi and sexual dysfunction only for male patients.

## Discussion

In Greek mythology the goddess Ariadne handed a clew of thread to Theseus to guide his way back out of the Minotaur's labyrinth. In the labyrinth of multimorbidity we also depend on a guide to find our way. As we have no other clue (or: clew), we must rely on statistical methods to explore the different pathways, i.e. the chains of associations in the pool of chronic conditions.

We made the following assumptions on the associations between the diseases: We supposed that there is a limited number of multimorbidity patterns (i.e. clusters of diagnosis groups that are significantly associated with each other). We expected that some diseases are associated with other diseases, while some diseases are rather independent of other diseases. We also assumed that some diseases are part of more than one pattern.

Previous research on multimorbidity patterns used cluster analyses to identify morbidity patterns [11]–[13]. At a given hierarchical level however, this cluster analysis would assign each disease to only one cluster, while in reality some diseases may be frequently associated with different patterns. In our data set this applies to seven diagnosis groups, i.e. depression, dizziness, renal insufficiency, atherosclerosis/peripheral arterial occlusive disease, anemias, chronic stroke and cardiac insufficiency. For this reason a cluster analysis of diseases might have produced an artificial pattern structure. Instead, a factor analysis fitted better with our assumptions. For each disease we know the degree of association with each pattern, so that diseases may become part of one or even several patterns. If the diseases are rather independent, there is little factor loading to each pattern.

Usually, the analysis of multimorbidity patterns is based upon a multimorbid population. This did not seem to be appropriate for our approach. In the factor analyses based on tetrachoric correlations, the dichotomous diagnoses were recalculated into continuous variables (see methods chapter), i.e. the illness data used for the exploratory analyses also reflect predispositions for illnesses as assumed by correlations between diagnosis groups. If we had excluded persons not being multimorbid or having no diseases at all we might have overestimated correlations between diagnosis groups and therefore biased the correlation matrix. For this reason we decided to base the analysis upon all insurants in the age group 65+ independently of the persons' number of diseases.

Persons are usually assigned to factors by performing a cluster analysis with the persons' individual factor scores [14]. We deviated from this method, because we presumed that the complexity of overlapping patterns (cf. figures 2 and 5) may not adequately be expressed by a limited number of clusters (normally 2 to 4). Instead, we assigned patients to a pattern if they had a diagnosis in at least three pattern-specific groups (cf. section statistical analyses). In doing so, we were also able to calculate prevalence rates for multimorbidity patterns and show how often these patterns overlap in individual patients.

### Strengths and weaknesses of the study

This study is the first to apply a factor analysis for identifying multimorbidity patterns. The factor analysis did perform well with the given data set. We had a limited number of three factors for both genders, a good model fit expressed by a high rate of cumulative percent (75% and 78%, respectively) and a sufficient sampling adequacy (Kaiser-Meyer-Olkin measure of 0.84 and 0.85, respectively).

A definite strength of our approach relates to a comprehensive picture of chronic diseases in the individual patients. We included all highly prevalent chronic conditions (≥1% in the age group 65+) into our diagnosis groups. For that reason we are quite sure that our statistical model is adjusted for noticeable influences of confounding diagnoses that may bias our results.

Although accidental and transitory diagnoses were excluded, in some cases diagnoses may be imprecise, ambiguous or incomplete because they were not clinically verified by trained professionals. This is a general problem in insurance claims data, but in our view, the benefits of claims data outweigh their disadvantages: We are provided with a large unselected population, representing real-world conditions and including persons living in protected institutions/nursing homes as well as frail individuals and the oldest olds, all frequently not included in survey and field studies. In choosing insurance claims data, we also avoided selection bias concerning service providers and as a matter of course there is no recall bias concerning diagnosis data.

### Patterns

We found three matchable multimorbidity patterns in both genders. Altogether 50.3% of female and 48.2% of male persons in our sample at least belong to one multimorbidity pattern. As the patterns differed only in singular diseases between men and women (see below) they are presented together.

The first pattern includes cardiovascular and metabolic disorders, an association that has long been known and became more clearly defined in the 1980s, as the term “metabolic syndrome” was established to designate the cluster of risk factors and diseases that come together in a single individual. The main features include insulin resistance (precursor of diabetes), hypertension and obesity [15], all of these conditions are found in this pattern. Gout was recently shown to be associated with the metabolic syndrome as well [16]. This coherence can be interpreted as an example for causal comorbidity, as these diseases probably tend to co-occur because they share the same risk factors (e.g. diabetes and gout [17]).

The second pattern found in our study covers anxiety, depression, somatoform disorders and pain. Groups of symptoms described in this pattern have appeared under different labels like “food intolerance,” [18] “chronic pain syndrome” [19] or "medically unexplained symptoms," [20] meaning that a definite medical diagnosis explaining the symptoms is often not established and a reasonable organic explanation is lacking [21]. While some of the diseases are clearly psychogenic (e.g. anxiety or depression) or clearly organic (e.g. arthrosis or osteoporosis), some can be assigned to mental and/or somatic causes, such as chronic back pain, gastritis or migraine. The association of anxiety, depression and somatic symptoms displayed in this pattern is well described [21]. A depressive or anxiety disorder is reported in about 30% of patients presenting physical complaints [22]. Others also report a close connection between anxiety, depression and gastrointestinal symptoms like gastritis or intestinal diverticulosis [23].

As a third pattern we found a group of diseases mainly consisting of neuropsychiatric disorders. Most combinations can be explained by causal comorbidity, such as dementia and Parkinson's disease probably being the causes for urinary incontinence. Other disease connections are more complex, for example cardiac insufficiency is a risk factor for stroke [24] which increases the risk for vascular dementia [25]. Another intricate association may result in the presence of anemia in the female pattern of neuropsychiatric disorders. There is an increased risk of carotid atherosclerosis and stroke (and therefore vascular dementia) in patients with renal insufficiency [26], which may also lead to anemia as a typical result of reduced kidney function [27].

The large overlapping of neuropsychiatric and cardiovascular/metabolic disorders (cf. figures 3 and 6) may also be explained by the relation between cardiovascular disorders, stroke and dementia. In addition, the pattern of neuropsychiatric disorders shows interference with the ADS and pain pattern. This correlation is well described, e.g. depression is a frequently described comorbidity in people after stroke [28] as well as patients with Alzheimer's disease or other forms of dementia [29].

### Age- and gender-specific differences in patterns

The prevalence of all patterns rises with the age of the patients. As age per se is the major risk factor for cardiovascular diseases [30] and metabolic syndrome [31], it is not surprising that the prevalence of this pattern strongly increases with age. Also, the prevalence of the pattern with neuropsychiatric disorders gains with increasing age as expected and already reported elsewhere [32].

Interestingly, there seems to be much less age dependency in the pattern of anxiety, depression, somatoform disorders and pain. This might in part be explained by underdiagnosis in older age, e.g. because older patients with clinically significant mental disorders tend to underreport their symptoms [33] or because the focus of doctors might move to manifest somatic diagnoses with increasing age of patients [34].

There are considerable differences in the composition of the patterns between the genders. The biggest differences can be found in the female pattern of neuropsychiatric disorders, which includes pre-terminal conditions such as chronic ischemic heart disease, renal insufficiency and anemia, suggesting an association of neuropsychiatric disorders and frailty in female patients. This might also explain the larger growth of this pattern with age in females than in males.

Gender differences are not always easy to explain. On one hand, a part of the differences between male and female patterns belongs to gender-specific morbidity as prostatic hyperplasia and sexual dysfunction in the male and non-inflammatory gynecological problems in the female ADS and pain pattern. On the other hand, gender differences in prevalence rates might to some extent account for the different composition of the patterns, e.g. rheumatoid arthritis exclusively belongs to female pattern of ADS and pain and has a prevalence in our sample of 3.9% in female, but only 1.7% in male patients and tobacco abuse exclusively belongs to the male cardiovascular/metabolic pattern and has 1.6% in male patients, but only 0.9% in female.

### Comparison with other studies

Three other research groups also made efforts to find clusters of multimorbidity. The results are quite diverse due to differences in study design and inclusion criteria: The studies differ in data sources (i.e. administrative data [11], survey data [12] and data from clinical examinations [13]), populations (e.g. US Veterans [11] or American Indian elders [12]) and number and type of diagnosis groups (i.e. 10 conditions including tuberculosis [12], 15 condition including hip fracture [13] or 23 conditions including HIV, post traumatic stress disorder and schizophrenia [11]). As mentioned above, all three studies conducted a cluster analysis.

Despite these differences in approach, there are still some common results in these studies. All studies report a similar cluster of cardiovascular and related diseases, in one case combined with metabolic disorders [11] like in our study, in another case combined with stroke [12] which was also found for male patients in our study. One other group also found an anxiety/depression cluster [11], but without the associated somatic disorders we found in our pattern. The other two studies did not include psychiatric disorders at all [12] or only in the form of depression [13]. Two studies included a neuropsychiatric cluster, one combined with peripheral vascular disease and seizures [11], another combined dementia, depression and hip fracture [13].

In an overall view, our results fit well with previous evidence. There are some minor discrepancies to our study, but they can be explained well by a new approach of identifying multimorbidity patterns, an unselected patient group and a more comprehensive list of included diagnosis groups in our study.

### Conclusions

The underlying structure of the labyrinth of multimorbidity sharpens if we allow for a little more complexity. As stated in our predetermined hypotheses the single multimorbidity patterns seem to share some diagnosis groups, to influence each other and to overlap in a large part of the population. We accommodated with these hypotheses by choosing a factor analysis based on tetrachoric correlations as our method. Also, it was important to base the exploratory analysis on an unselected population and a comprehensive selection of diagnosis groups to avoid blurring the subtle ramifications of the labyrinth.

Our clew of thread leads us through three prevalent pathways of multimorbidity, i.e. clusters of statistically significant co-occurrence of chronic diseases. About 50% of all persons of 65 years and older belong to at least one multimorbidity pattern. The patterns of cardiovascular/metabolic, neuropsychiatric and anxiety/depression/somatoform disorders and pain fit well with existing evidence. Research is still needed concerning the impact of the different patterns. Future studies should especially focus on interactions between the patterns and (negative) synergy effects of multiple patterns in individual patients. In recognizing the full complexity of multimorbidity we might improve our ability to predict needs and achieve possible benefits for elderly patients who suffer from multiple chronic conditions.
